# Dr. Badri Teymourtash (1908–1995), and her Key Role in Foundation of Mashhad Dental School in Northeastern Iran

**DOI:** 10.34172/aim.34229

**Published:** 2025-07-01

**Authors:** Hediyeh Toutouni, Shirin Taraz Jamshidi, Ali Emadzadeh

**Affiliations:** ^1^Community Oral Health Department, Mashhad Dental School, Mashhad University of Medical Sciences, Mashhad, Iran; ^2^Department of Pathology, Faculty of Medicine, Mashhad University of Medical Sciences, Mashhad, Iran; ^3^Department of Internal Medicine, MMS.C, Islamic Azad University, Mashhad, Iran

**Keywords:** Dentistry, History, Iran

## Abstract

Doctor Badri Teymourtash (1908‒1995) was among the first Iranian lady dentists. She was one of the founders of Mashhad Dental School and one of the pioneers of modern dentistry in Iran. This paper takes a glance at the story of the life of this academic woman with its ebbs and flows.

## A Little Girl from Nardin

 In 1908 AD, in Nardin, a remote village in Northeastern Iran, a little girl was born who became one of the most important and impressive persons in the history of dentistry in Iran.^1,2^ Her parents named her Badri.Nardin, a small village located in the Khorasan province at that time (now part of the Semnan province in the new categorization of provinces in Iran) was ruled by landholders, like other parts of Iran. Badri’s father, Karimdad Khan Nardini, the great landholder of Nardin, was the governor of Sabzevar in the era of Muzaffar Al-din Shah Qajar,^3^ and so Badri was raised in an aristocratic and wealthy family. Badri’s mother, Gowhartaj Khanum, the last wife of Karimdad Khan, had no child but Badri.^2^ Badri had numerous half-brothers and half-sisters.^2^ Her older half-brother, Abdolhossein Teymourtash, was a famous Iranian politician; a fact which later affected Badri’s life, as mentioned below.

**Figure 1:**
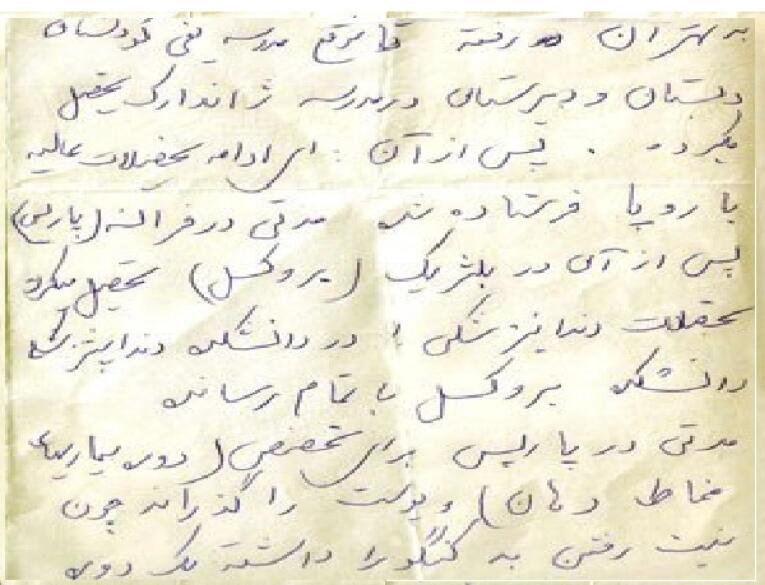
A Part of Dr. Badri Teymourtash’s Manuscript About Her Biography, Written in May 1991 (Courtesy of Ms. Azar Vakili)

 Badri completed her primary and secondary educations at Jeanne d’Arc School, in Tehran, capital of Iran (Figure 1).^4^ After a couple of years, she was sent to Paris, France, and then to Brussels, Belgium, in the early 1930s to study dentistry.^1,2^ She decided to move to Belgian Congo after her university graduation to participate in charity plans, but the storm of political unrest in her homecountry, Iran, even affected Badri, thousands of kilometers away from Iran, in Belgium.

## Who Was AbdolhosseinTeymourtash?

 Abdolhossein Teymourtash, Badri’s elder brother, was born in 1881,^3^ so he was 27 years older than his little sister. After their father’s death, Abdolhosein took care of her little sister. He, as one of the most impressive political Iranian men, was one of the most important persons who had a role in the transition of power from the House of Qajar to the Pahlavi dynasty in 1925.^3,5^ He was elected as the Minister of the Royal Court in the era of Reza Shah Pahlavi between 1925 and 1933.^3^ There are different points of view about his personality and his political acts, the discussion of which is beyond the scope of this article and this journal, and the readers are referred to the huge bulk of papers and books written about him^3,5-8^ After about 8 years playing a significant role in Iranian diplomacy, he was dismissed from office by Reza Shah.^3^ He was arrested and imprisoned in Qasr Prison in Tehran.^3^ Soon after, his health progressively deteriorated, and he died on October 1, 1933.^3^ Many believe that he was killed by the prison’s physician, Dr. Ahmad Ahmadi, through lethal air injections.^3^ After Abdolhossein Teymourtash’s death, his properties were impounded by the government, while his first-degree family members were exiled to a remote village around Kashmar in the Khorasan Province, northeastern Iran.^2,3^ In the case of Badri, for instance, when her university tuition, which was regularly sent from Iran, was discontinued and she had to struggle with poverty, she was commanded back to Iran.^2^ Along with her mother and other family members, she spent seven years in exile.^2^ After Reza Shah’s abdication in September 1941 and the announcement of general amnesty, the members of the Teymourtash family were released from exile.^2,3^

## Release from Exile, Moving to Mashhad, and the Foundation of Mashhad Dental School

**Figure 2:**
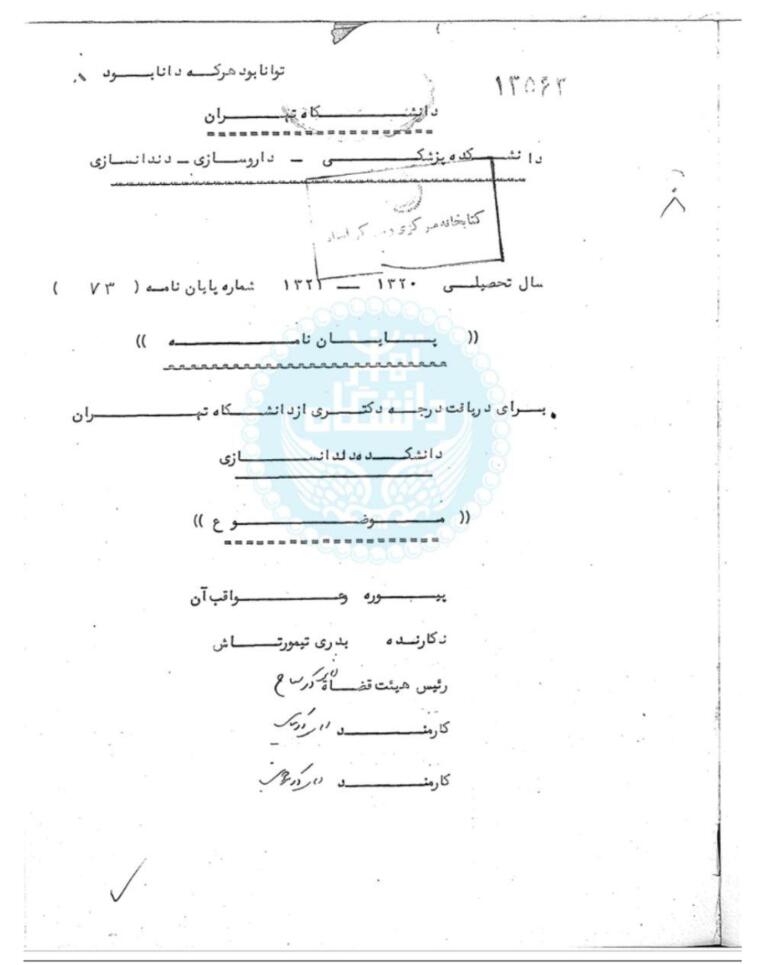
The First Page of Dr. Badri Teymourtash’s Dissertation. Adapted from Mirsaeidi, with permission^9^

 Having been released from exile, Badri completed her education at Tehran University. Some believe she completed a few remaining dentistry courses at the Faculty of Dentistry of Tehran, but others believe that she only did her dissertation at Tehran University.^9^ Her dissertation’s title was “Pyorrhea and its complications” and it was performed under the supervision of Dr. Mohsen Sayyah in the scholar year 1941‒42 (Figure 2).^9^ She dedicated her dissertation to her brother, Abdolhossein, and her nephew, Mehrpour.^9^ That dissertation consisted of 90 pages including five separate parts.^9^

**Figure 3:**
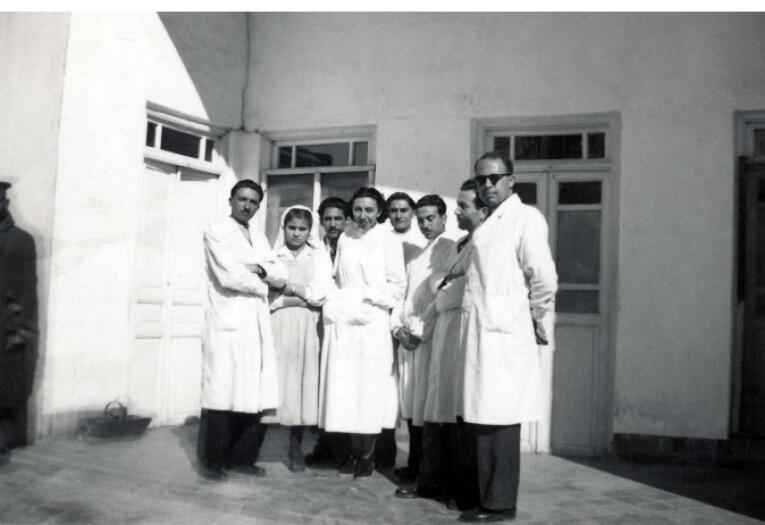
Dr. Badri Teymourtash, surrounded by the nurses and other staff of Imam Reza Hospital, probably in the early 1950s, before the foundation of Mashhad Dental School, Adapted from Dr. Masoumeh Khazaei’s album, with permission.

 While in exile, Dr. Badri made a vow that after release from exile, she would transfer to Mashhad.^2^ After a couple of years, she kept her promise and moved to Mashhad in the mid-1940s.^1^ Mashhad, located in northeastern Iran, is the most populous and largest Iranian city after the capital, Tehran, as well as a holy city for Shia Muslims as it is home to the Holy Shrine of Imam Reza, the 8^th^ religious leader of Shias. When Badri started her new life in Mashhad, she worked as a dentist in her private clinic and in an office at Imam Reza Hospital.^1,2^ Imam Reza Hospital (formerly called Shah Reza Hospital) was the main educational hospital of Mashhad University. In those years, Imam Reza Hospital was the host of distinguished expert physicians from Iran and the western European countries which Astan Quds Razavi (A.Q.R.) invited to empower the medical staff of Mashhad University.^10^ She also taught at the medical school of Mashhad (Figure 3).^2^ After a few years, she separated the dental school from the medical school. Finally, she and Dr. Amir Esmaeil Sondouzi succeeded in opening the School of Dentistry of Mashhad University in 1965.^11^ This school was the second dental school in Iran to award the university degree of Doctor of Dentistry (DDS) to its graduates within the higher education platform.^11^

**Figure 4:**
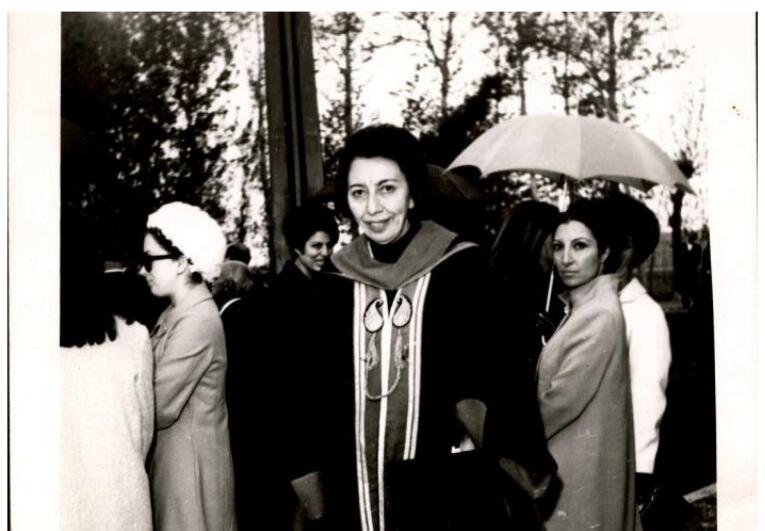
Dr Badri Teymourtash in Manba’Garden, the First Location of the School of Dentistry of Mashhad, after Obtaining Associate Professorship of Mashhad University, Late 1960s (Adapted from Ms. Azar Vakili’s Album, with permission)

 After Dr. Fereydon Farzin relocated to Shahid Beheshti University (formerly called Melli University) in Tehran in 1967, Dr. Badri Teymourtash was elected as the dean of Mashhad Dental School.^2,11^ It was the first time that a woman assumed such a position in Iran (Figure 4).^11^

**Figure 5:**
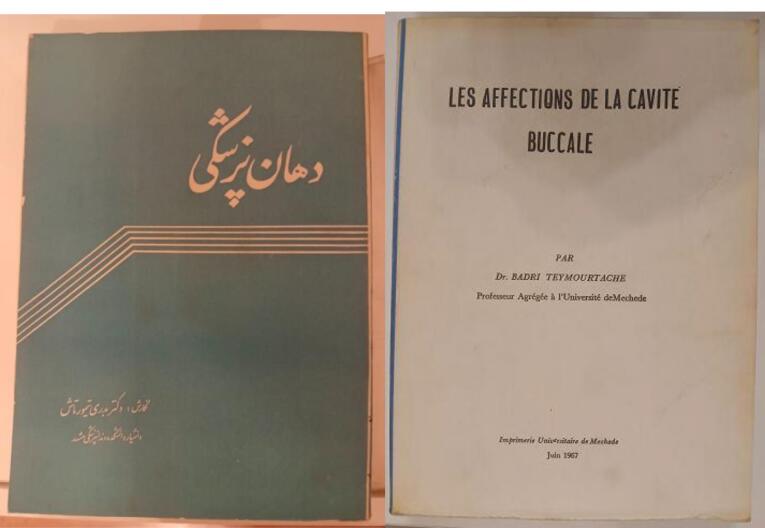
The Front Cover and Back Cover of the Academic Textbook “Dahaan Pezeshki” [in Persian] “Oral Medicine / Les Affections de la Cavite Buccale”, Compiled by Dr. Badri Teymourtash, Published by Ferdowsi University of Mashhad. June 1967. Courtesy of Dr. Mahshid Bahadori

 Soon after the establishment of the School of Dentistry, she closed her private clinic, because she devoted her life to training dental students and did not have enough time to manage the school’s educational and administrative affairs.^2^ Besides training dentistry students, she compiled some academic textbooks, among which “*Dahaan Pezeshki*” (Oral Medicine) is highly valuable (Figure 5).

## Personal Life

 Dr. Badri never married.^2^ In the mid-1960s, she received a 6-year-old child, Azar Vakili, as her goddaughter.^1,2^ After Azar left her parents, she became the best friend of her godmother for the remaining three decades of Dr. Badri’s life.

**Figure 6:**
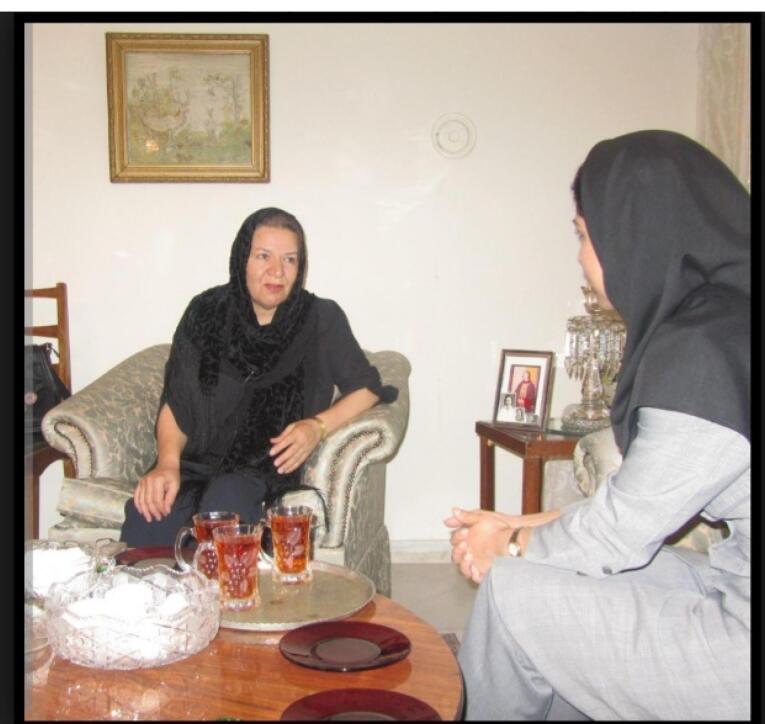
Azar Vakili, Dr. Badri Teymourtash’s goddaughter, spends her retirement time with the 30 years of life with her godmother, Dr. Badri Teymourtash. The first author of this paper interviewing Ms. Azar Vakili in her home, Mashhad, Iran

 Dr. Badri was very generous, helped her students and patients overcome their financial problems, and was hospitable to every person who entered her home as a guest.^2^ Under the guidance of Dr. Badri, Azar entered Mashhad Nursing School, and became one of that school’s academic staff. Nowadays, Azar spends her retirement time with the memories of 30 years of life with her godmother, Dr. Badri Teymourtash (Figure 6).^2^

## Aftermath

**Figure 7:**
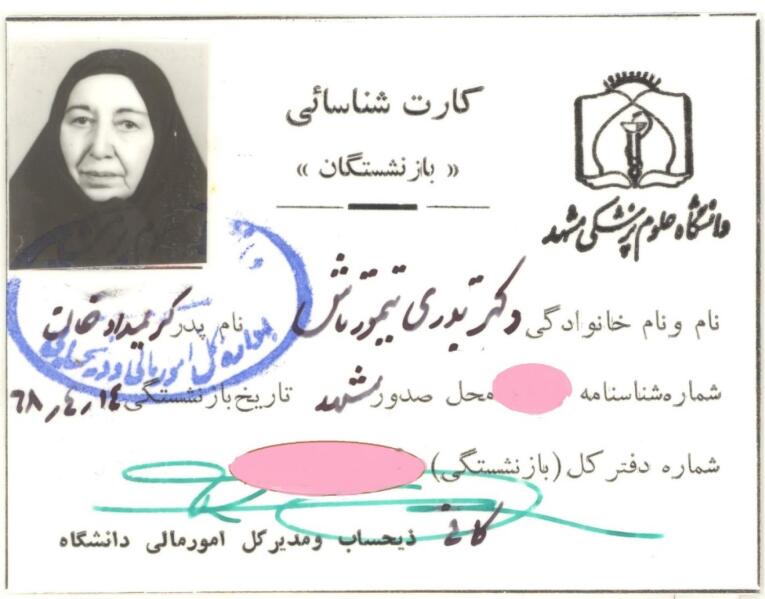
Dr. Badri Teymourtash’s ID Card after her Retirement. Adapted from Ms. Azar Vakili’s album, with permission

**Figure 8:**
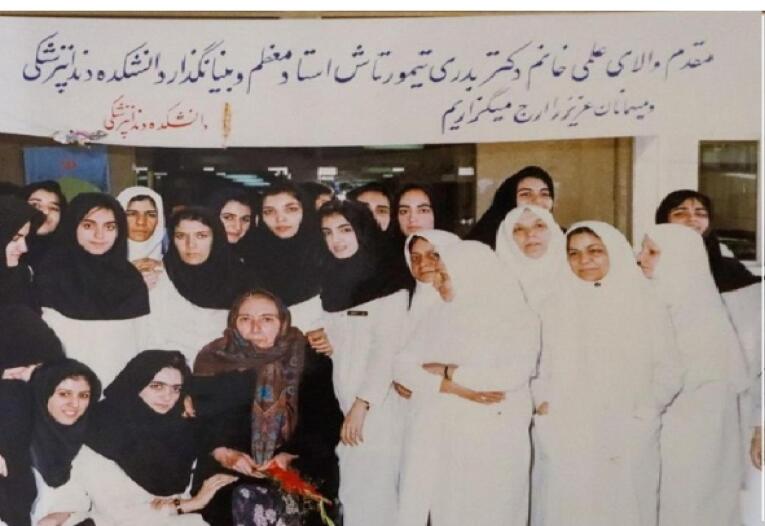
Dr. Badri Teymourtash, Surrounded by the Staff of Mashhad Dental School, the Ceremony of Teacher’s Day, 2 May 1991. Adapted from Dr. Maryam Khurdi Moud’s collection, with permission

**Figure 9:**
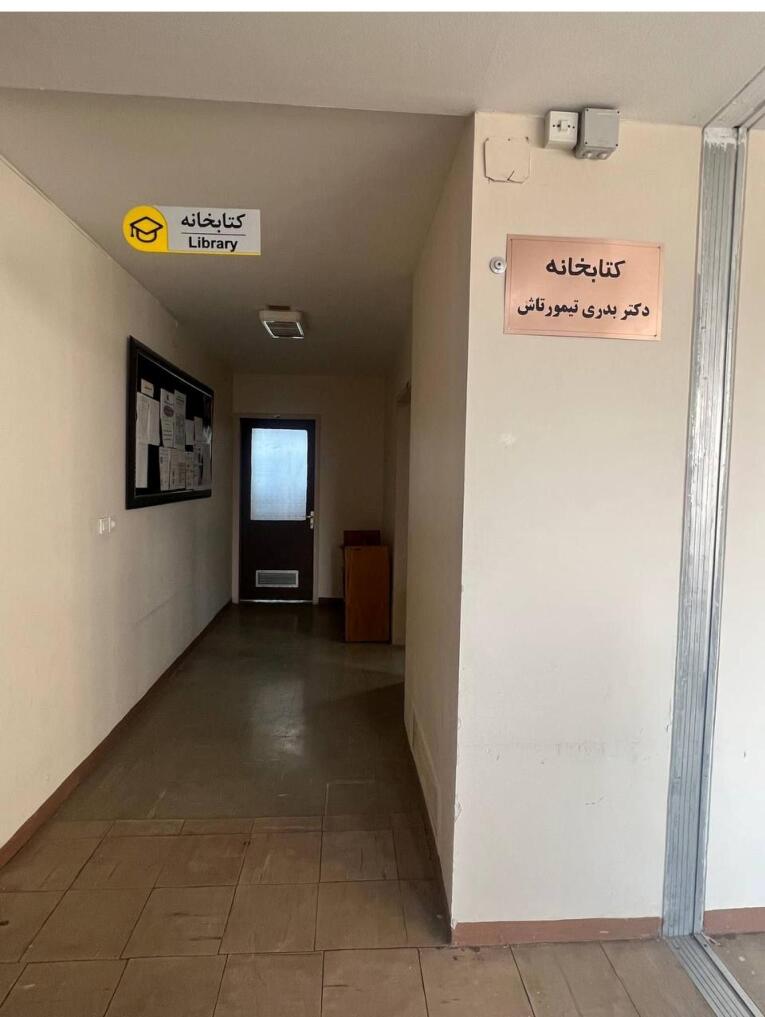
The Library of Dr. Badri Teymourtash, Mashhad Dental School, photo taken by the first author

 In 1972, after her mother passed away, she became depressed and spent more time with her goddaughter, Azar.^2^ In 1989, she retired and seldom left her house (Figure 7).^2^ In May 1991, in a glorious ceremony, the School of Dentistry of Mashhad’s Library was named “Library of Dr. Badri Teymourtash”, in honor of her efforts for the foundation of that school. (Figures 8 and 9).^2^ She delivered an interesting speech on that occasion.^1,2^ That was the last time Dr. Teymourtash participated in an academic event.^2^

**Figure 10:**
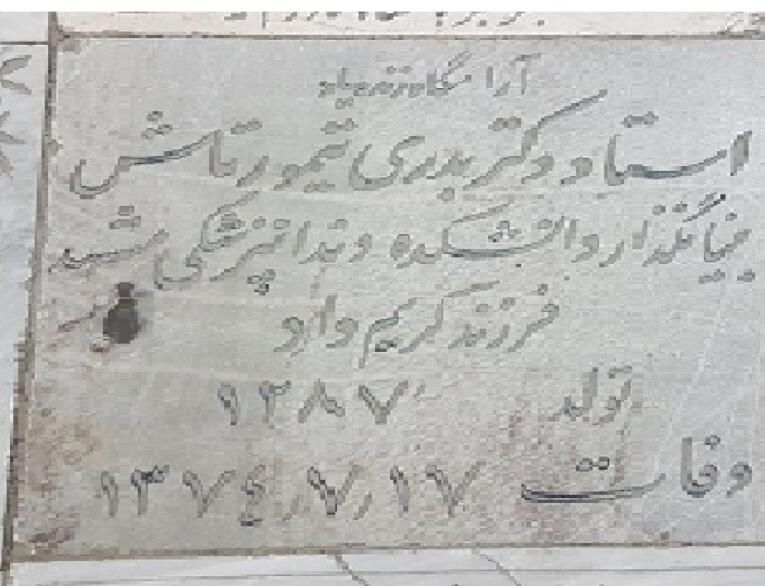
The Tombstone of Dr. Badri Teymourtash, Azadi Courtyard, Imam Reza Holy Shrine, Mashhad, Iran, photo taken by the first author

 In the summer of 1995, she suffered a severe stroke, and after about two months in a comatose state, she passed away on Monday, Oct. 9, 1995. She was buried in the Azadi Courtyard at Imam Reza Holy Shrine in Mashhad^2^ (Figure 10).

## Legacy

 Although she was not the first Iranian female dentist, she was the first Iranian woman who founded a dentistry school and the first woman to hold the position of the dean of such a school in Iran. The members of the medical sciences community of Iran always remember her, not only because of her impressive role in the foundation of the second Dentistry School in Iran, but also for her generosity, kindness, and playing a maternal role for her students. Studying the history of the life of an aristocratic girl who tried so much to achieve a high academic degree, grabbed with poverty, suffered an extended period in exile without any guilt, and performed much effort for training students, can be a bright light for our road in life. It seems that her legacy is not only a name on a table at the entrance of a library (Figure 9), but her main heritage is the foundation of an organization that trained numerous generations of dentists who are considered her spiritual children.
